# Fast motif recognition via application of statistical thresholds

**DOI:** 10.1186/1471-2105-11-S1-S11

**Published:** 2010-01-18

**Authors:** Christina Boucher, James King

**Affiliations:** 1 David R. Cheriton School of Computer Science, University of Waterloo, Waterloo, Ontario, Canada; 2 School of Computer Science, McGill University, Montreal, Quebec, Canada

## Abstract

**Background:**

Improving the accuracy and efficiency of motif recognition is an important computational challenge that has application to detecting transcription factor binding sites in genomic data. Closely related to motif recognition is the CONSENSUS STRING decision problem that asks, given a parameter *d *and a set of ℓ-length strings *S *= {*s*_1_, ..., *s*_*n*_}, whether there exists a consensus string that has Hamming distance at most *d *from any string in *S*. A set of strings *S *is *pairwise bounded *if the Hamming distance between any pair of strings in *S *is at most 2*d*. It is trivial to determine whether a set is pairwise bounded, and a set cannot have a consensus string unless it is pairwise bounded. We use CONSENSUS STRING to determine whether or not a pairwise bounded set has a consensus. Unfortunately, CONSENSUS STRING is NP-complete. The lack of an efficient method to solve the CONSENSUS STRING problem has caused it to become a computational bottleneck in *MCL-WMR*, a motif recognition program capable of solving difficult motif recognition problem instances.

**Results:**

We focus on the development of a method for solving CONSENSUS STRING quickly with a small probability of error. We apply this heuristic to develop a new motif recognition program, *sMCL-WMR*, which has impressive accuracy and efficiency. We demonstrate the performance of *sMCL-WMR *in detecting weak motifs in large data sets and in real genomic data sets, and compare the performance to other leading motif recognition programs. In our preliminary discussion of our CONSENSUS STRING algorithm we give insight into the issue of sampling pairwise bounded sets, and discuss its relevance to motif recognition.

**Conclusion:**

Our novel heuristic gives birth to a state of the art program, *sMCL-WMR*, that is capable of detecting weak motifs in data sets with a large number of strings. *sMCL-WMR *is orders of magnitude faster than its predecessor *MCL-WMR *and is capable of solving previously unsolved synthetic motif recognition problems. Lastly, *sMCL-WMR *shows impressive accuracy in detecting transcription factor binding sites in the genomic data and used in the assessment of Tompa *et al*.

## Background

Given a number of DNA strings, *motif recognition *is the task of discovering similar substrings without prior knowledge of their consensus or their locations. The following is a combinatorial formulation of the (ℓ, *d*)-motif problem [1]: let *S *= {*s*_1_, ..., *s*_*n*_} be a set of *m*-length strings, and s* be the *consensus string*, a fixed and unknown string of length ℓ that is contained in each *s*_*i *_as a substring but is corrupted with at most *d *substitutions (point mutations). The aim is to determine s* and the location of the motif instances in each string. The *weak motif recognition problem *is to find the motif instances when the number of degenerate positions *d *is large in relation to the motif length ℓ; well-known weak motif recognition problems exist when the parameters (ℓ, *d*) are equal to (11, 3), (15, 4), and (18, 6). This combinatorial problem has application to finding transcription factor binding sites in genomic data [2].

Motif recognition is NP-complete and therefore cannot be solved in polynomial time unless P = NP [3]. Nonetheless, there are numerous algorithms developed to solve specific instances of the problem, including PROJECTION [4], Winnower [1], pattern driven approaches [5], MITRA [6], PSM1 [7], PMSprune [8], the Voting algorithm [9], MCL-WMR [10], MEME [11], VAS [12], RISOTTO [13], Weeder [14] and several others. Li *et al*. proved the existence of a PTAS for an optimization version of the motif recognition problem, though the high degree in the polynomial complexity of the PTAS algorithm renders this result only of theoretical interest [15].

Closely related to motif recognition is the CONSENSUS STRING decision problem. A consensus string for a set *S *of strings has Hamming distance at most *d *from all strings in *S*. CONSENSUS STRING asks, given a parameter *d *and a set *S *= {*s*_1_, ..., *s*_*n*_} of *n *strings, each of length ℓ, whether there exists a consensus string for *S*. CONSENSUS STRING is NP-complete even when interest is limited to the binary alphabet [16].

For a given parameter *d *we say *S *is a *motif set *if there exists a consensus string s* at distance at most *d *from any string in *S*; we say a set *S *of strings is *pairwise bounded *if the distance between any pair of strings in *S *is at most 2*d*. Every motif set is pairwise bounded; if a pairwise bounded set is not a motif set we say it is a *decoy set*. For example, for *d *= 1 the set {000, 001, 010, 100} is a motif set because 000 is a consensus string for this set. In contrast, the set {000, 011, 101, 110} is a decoy set because it is pairwise bounded (since any two of the strings are at Hamming distance 2) but no consensus string exists.

The focus of this paper is the development and application of a heuristic for the CONSENSUS STRING decision problem (also known as the RADIUS DECISION problem [16]). We denote the Hamming distance between any pair of strings *s*_*i *_and *s*_*j *_as *H*(*s*_*i*_, *s*_*j*_). We define the *weight *of a set of strings *S *as the sum of the Hamming distances of each pair of strings in *S *(*i.e*. Σ_1 ≤ *i *≤ *j *≤ *n *_*H*(*s*_*i*_, *s*_*j*_)). If the *weight *of a set, which can be calculated in polynomial time, can be used to indicate whether it is a motif set or a decoy set then CONSENSUS STRING can be solved extremely efficiently and accurately in practice--simply calculate the weight of the pairwise bounded set and decide whether the set has a consensus based on this value. For this heuristic to work we need to know how the respective weights of a random motif set and a random decoy set are distributed. Further, the distributions need to be adequately separated so that the weight of a set leaves little ambiguity as to whether the set is a motif set or a decoy set.

There exists an algorithm to sample from the set of all motif sets: simply choose any ℓ-length string as the consensus sequence and sample with replacement from the set of all strings that are at distance at most *d *from that sequence [10]. Unfortunately we do not know an analogous sampling algorithm, either exact or approximate, for decoy sets. If we could sample pairwise bounded sets uniformly then we could learn the probability distribution of the weight of a random decoy set.

We give a method to generate pairwise bounded sets uniformly, use this method to determine the probability distribution of the weight of a random decoy set, and show the existence of a separation between this distribution and the probability distribution of the weight of a random motif set. Thus, we solve CONSENSUS STRING instances extremely accurately and efficiently using the simple heuristic of using the weight as an indicator as to whether a pairwise bounded set is a motif set or a decoy set. The separation of the distributions becomes increasingly more prevalent as the number of strings in the set (*i.e*. the parameter *n*) increases, so the accuracy of our method increases as the number of strings increases. We significantly extend our earlier motif recognition program, *MCL-WMR *[10], by incorporating the heuristic for CONSENSUS STRING described in this paper. This new algorithm, referred to as *sMCL-WMR*, detects motifs in data sets with a large number of strings (*i.e*. 30 or more strings), and finds regulatory strings in genomic data. sMCL-WMR represents the input data as a weighted graph and uses graph clustering to narrow the search to smaller problems that can be solved with significantly less computation. An efficient refinement algorithm that distinguishes valid motif sets from decoy sets allows sMCL-WMR to detect motifs in very large data sets in significantly less computational time than MCL-WMR.

## Methods

### Sampling pairwise bounded sets

In this section we discuss uniform sampling, or generation, of pairwise bounded sets. A standard method used to generate a random motif set is to choose an ℓ-length string u.a.r. (uniformly at random) from all possible 4^ℓ ^strings to be the consensus string, and then form a motif set by selecting *n *strings at random with replacement from the set of all strings with Hamming distance at most *d *from this consensus string [4,10]. This does not sample motif sets uniformly, but rather samples a motif set with probability proportional to the number of distinct consensus strings it has and thus, corresponds to how synthetic problem data sets are constructed and how we expect meaningful motif sets arise in nature. For example, synthetic problem instances are traditionally generated as follows: a random consensus string of length ℓ is chosen, *n *occurrences of the motif are generated by randomly mutating at most *d *positions, and each of the *n *motif instances is embedded at a random location into a different background string of length *m*. We note that other non-uniform distributions have also been used to generate motif sets [1].

When sampling uniformly from a poorly understood sample set, *rejection sampling *is a naïve but useful technique. If we can find a superset of the target set that is easy to sample from uniformly, we can sample from this superset and simply throw away (reject) any sampled element that is not in the target set. We show how rejection sampling can be applied to generate pairwise bounded sets uniformly.

#### Uniform sampling of pairwise bounded sets

To sample u.a.r. from all pairwise bounded sets using rejection sampling in the most naïve way, we would generate *n *random ℓ-length strings and accept the set if it is pairwise bounded, and reject and repeat otherwise (technically this samples uniformly from pairwise bounded *sequences *since the order of the strings matters in a sequence). However, since it is unlikely that such a randomly generated set would be pairwise bounded, this method is extremely inefficient. We introduce a heuristic to generate random sets that are more likely to be pairwise bounded, thus speeding up the rejection sampling process enough to be practical.

We generate the first string, *s*_1_, u.a.r. from the set of all ℓ-length strings then generate each of *s*_2_, ..., *s*_*n *_in turn u.a.r. from the set of all strings at distance at most 2*d *from *s*_1_. This gives us a set of strings generated u.a.r. from the set of all strings that have *s*_1 _as the first string and each other string at distance at most 2*d *from *s*_1_. If the set is pairwise bounded we keep it; if it is not we reject it and start over. The fact that this method generates pairwise bounded sequences u.a.r. can be verified by induction on *n*. The number of times a set of *n *strings is considered and rejected until a pairwise bounded set is generated follows a geometric distribution and therefore, the efficiency of this method is determined by the probability that a set is rejected. Though this method is fast enough to work in practice for values of *n *we are interested in, the expected runtime when generating a single pairwise bounded set grows exponentially with *n*.

**Proposition 1**. *The probability that a set generated using rejection sampling is pairwise bounded decreases at least exponentially fast as a function of n*.

Proof. For 1 ≤ *i *≤ *n *let *S*_*i *_be the subset of *S *containing the first *i *randomly chosen strings, with *S*_*n *_= *S*. Let *A*_*i *_be the event that *S*_*i *_is pairwise bounded. Any subset of a pairwise bounded set is pairwise bounded, so *A*_*i *_implies A_*i*-1 _for 2 ≤ *i *≤ *n*. Therefore by Bayes' law we have ℙ[*A*_*i*_] = ℙ[*A*_*i*_|*A*_*i*-1_] ℙ[*A*_*i*-1_]. To prove that ℙ[*A*_*n*_] decays exponentially with *n *we need only show that ℙ[*A*_*i*_|*A*_*i*-1_] is non-increasing in *i*, since it can easily be verified to be strictly less than 1 for *i *= 3. Let *K*_*i *_be the set of strings such that *S*_*i *_∪ {*s*} is pairwise bounded if and only if *s *∈ *K*_*i*_, noting that *K*_*i *_= ∅ if *S*_*i *_is not pairwise bounded. We have *K*_*j *_⊆ *K*_*i *_for any 1 ≤ *i *<*j *≤ *n*. Since , where *B*(2*d*) is the number of strings at distance at most 2*d *from *s*_1_, the result holds.   □

To empirically evaluate the efficiency of our rejection sampling method we determined the portion of sets that will be rejected when generating a sample (of specified size) of pairwise bounded sets. We performed experiments with varying values of *n*, ℓ, and *d*, generated 10000 pairwise bounded sets in each experiment, and considered the average number of sets rejected before the pairwise bounded set was obtained. The default values for (*n*, ℓ, *d*) are (20, 15, 4).

**Figure 1:**
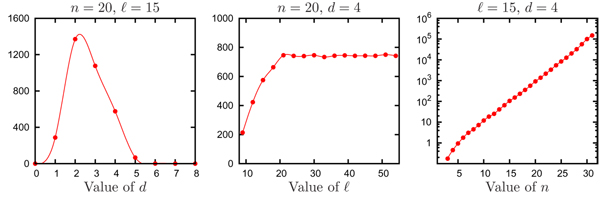
**Efficiency of rejection sampling**. Average number of rejections when generating a pairwise bounded set with our rejection sampling heuristic. Each plot shows the effect of varying one of the three parameters (*n*, ℓ, *d*). Data points are connected with cubic splines. Note the logarithmic scale used in the right plot.

The results of the empirical tests are shown in Figure 1. Each of the three plots shows how the average number of rejected sets changes when one of the three parameters is varied and the other two are fixed at their default values. The left plot shows what happens when *d *varies between 1 to 7. For values of *d *that are either greater than ⌊ℓ/2⌋ or equal to 0, any set we generate is pairwise bounded and hence, we did not plot data for *d *= 0 or *d *≥ 8. The average number of rejected sets is largest when *d *is equal to 2 and decreases dramatically as *d *increases. This trend is expected since a large portion of non-pairwise bounded sets would be rejected when *d *is moderately large. The middle plot shows what happens when ℓ is varied between 9 and 55. The number of rejected sets increases steadily when ℓ varies within the range [9,20], then plateaus when ℓ is above 20. It can be easily shown analytically that increasing ℓ above 2*dn *will have no effect, however, we see empirically that the effect of ℓ is minimal for values of ℓ greater than 20. The right plot shows the effect of varying *n *between 3 and 31. Noting that a logarithmic scale is used, the average number of rejected sets exhibits growth that is clearly exponential in *n*.

### A separation of weight distributions

**Figure 2:**
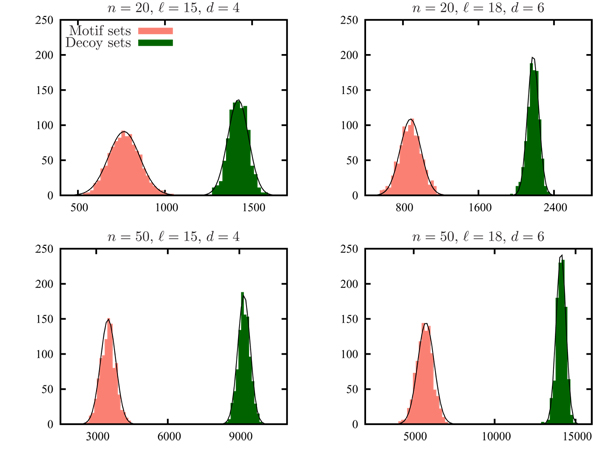
**Weight distribution histograms**. Histograms showing weight distributions for motif sets and decoy sets. Normal distributions fitted to the data are shown to indicate that the weight distributions are approximately normal.

One of the key motivations for the development of methods to generate pairwise bounded sets from an appropriate distribution is that it can be used to determine whether there is a separation between the probability distribution of the weight of a random valid motif set and that of a random decoy set. We use the sampling method just described to generate 1000 random motif sets and 1000 random decoy sets for varying values of (ℓ, *d*) and *n*. For each random motif and decoy set witnessed we calculated the weight of the set. Figure 2 depicts, for values considered for (ℓ, *d*) and *n*, the distribution of the weight of the 1000 random motif sets and that of the 1000 random decoy sets. The data illustrate an adequate separation between the distributions.

As the value of *n *increases, the separation between the distributions becomes more prevalent since the probability distributions become more concentrated around their means and the means themselves diverge. Further, the dichotomy is again more evident when (ℓ, *d*) is increased from (15, 4) to (18, 6). When *n *is even moderately large we can use the weight to determine accurately whether the set is a motif set or a decoy set and as *n *increases this method of using the weight as an indicator will likely increase in accuracy. Similar conclusions can be made when ℓ and *d *increase. These results suggest that the simple heuristic of using the weight to determine whether a pairwise bounded set is a valid motif set or a decoy set will enable computationally challenging instances of the CONSENSUS STRING problem (e.g. when *n *≥ 20 or (*l*, *d*) is equal to (18, 6)) to be solved efficiently with minimal probability of error.

These empirical trends illustrate the analytical results of Boucher *et al*. [10] that demonstrate that the distribution of the weight of a random motif set is tightly concentrated around its mean. The following theorem proves that the distribution of *W*_*m *_is sharply concentrated around its mean; specifically it provides exponential tail bounds.

**Theorem 1 (Strong concentration bound for motif sets **[10]). Let *W*_*m *_*be the weight of a random motif set and μ*_*m *_*be the expected value of W*_*m*_. *Then for any λ *> 0,

It is currently open to prove an analogous result to Theorem 1 for an arbitrary decoy set. This is a considerably more challenging problem due to the lack of a combinatorial characterization of a decoy set.

### sMCL-WMR: an efficient method to detect motifs in large data sets

In 2007, MCL-WMR was developed specifically for the problem of detecting weak motifs in genomic data [10]. One of the main contributions of MCL-WMR is the introduction of a novel weighted-graph model for motif recognition. Unfortunately, MCL-WMR was unable to detect motifs beyond when ℓ = 18, *d *= 6, *m *= 1000, and *n *≥ 20 [10]. Eskin and Pevzner reported similar results for various motif recognition programs [6], and Feng *et al*. showed limited accuracy for the (15, 4) problem with 20 strings of length 600 [17]. Specific motif recognition problems--that is, the problem for specific values of *n*, *m*, ℓ, and *d*--have remained intractable. For example, MCL-WMR was unable to solve any instance of the (25, 8) motif recognition problem with *n *= 20.

MCL-WMR uses graph clustering to determine pairwise bounded sets that might be valid motifs. The major impediment to the efficiency of MCL-WMR was the exponential-time refinement algorithm used to determine which "candidate motif sets" (*i.e*. pairwise bounded sets) have a consensus string [10]; this step becomes a bottleneck for solving challenging weak motif instances, such as (18, 6), when the number of such candidate sets increases dramatically [4]. Boucher and Brown [18] give a probabilistic heuristic for solving the consensus string problem, which filters candidate sets based on a "majority vote", that has acceptable accuracy when *n *is significantly large (*i.e*. when *n *≥ 20). We propose a probabilistic algorithm that eliminates the need for a strong bound on *n*; our novel algorithm uses a candidate set's weight to determine quickly and with a small probability of error whether the set is a decoy set or a motif set.

### Overview of system

sMCL-WMR considers a weighted graph representation of the data set, where each substring of length ℓ is represented by a vertex and the construction of our graph  ensures that the motif instances represented by vertices in the graph are connected to each other and form a clique of size n, though the converse need not hold. In this model, the problem of finding pairwise bounded sets in the data reduces to finding cliques of size *n *in the graph .

1. The vertex set contains a vertex *v*_*i*, *j *_representing the ℓ-length substring in string *i *starting at position *j*, for each *i *and *j *= 1, 2, ..., *m *- ℓ + 1. There are *n*(*m *- ℓ + 1) vertices.

2. Each pair of vertices *v*_*i*, *j *_and *v*_*i*', *j*'_, for *i *≠ *i*' is joined by an edge if and only if the corresponding substrings are at Hamming distance at most 2*d*.

3. An edge between vertices having distance *k *has weight ℓ - *k *for *d *<*k *≤ 2*d*, or 10(ℓ - *k*) for *k *≤ *d*. This emphasizes substrings at small distances.

We chose to use the *Markov cluster algorithm *(*MCL*) [19] to cluster the graph  due to its ability to handle large weighted graphs. We reduce the size of the instance being passed to MCL by considering subgraphs  = {*G*_1_, *G*_2_, ..., *G*_*m*-ℓ+1_}, where, for some arbitrary choice of reference string *R*, *G*_*j *_is the subgraph induced by the closed neighborhood of the reference vertex *v*_*R*, *j*_. This is more efficient than searching all of  at once. MCL then clusters each *G*_*i *_∈  to determine subgraphs that are highly interconnected (high edge weight within a cluster). A clique in *G*_*i *_that represents a pairwise bounded set must have size *n *and have weight at least (ℓ - 2*d*) since each pair of vertices must be adjacent. We filter out the clusters produced by MCL that do not meet these criteria since they cannot contain sufficiently large cliques. MCL-WMR uses a dynamic programming algorithm to determine which pairwise bounded sets (or cliques) represent valid motif sets; this computationally intensive step limits its ability to solve many motif recognition instances.

Figure 2 illustrates that both the weight of a random motif set and that of a random decoy set are approximately normally distributed, and shows a separation between these distributions. Using the rejection sampling method described earlier we calculate the mean and standard deviation of the weight of a random motif set and the weight of a random decoy set. We use *N*(*μ*, *σ*^2^) to denote a normal distribution with mean *μ *and variance *σ*^2^. Let random variables *W*_*m *_and *W*_*d *_denote the weight of a random motif set and the weight of a random decoy set, respectively. Let *μ*_*m *_and  respectively denote the mean and variance of the distribution of *W*_*m *_and similarly, let *μ*_*d *_and  respectively denote the mean and variance of *W*_*d*_. Assuming that *W*_*m *_~ *N *(*μ*_*m*_, ) and *W*_*d *_~ *N *(*μ*_*d*_, ), we can determine the values *α*_*m *_and *α*_*d *_such that:

If *α*_*m *_<*α*_*d *_then we can use the weight of a pairwise bounded set of strings to determine whether the set is a decoy or a motif as follows: calculate the weight *w *of the set and, if *w *≤ *α*_*m *_or *w *≥ *α*_*d *_then return that the set is a motif or a decoy, respectively; otherwise, use the dynamic programming algorithm to classify the set. Hence, if *α*_*m *_<*α*_*d *_then more than 99% of pairwise bounded sets will be classified correctly by considering the weight of the set. Typically the gap between *α*_*m *_and *α*_*d *_is large enough to guarantee that this rate is far higher than 99%. In theory it is possible that a set could be misclassified (*e.g. *if a motif set has weight greater than *α*_*d*_) though in practice the probability of this happening is negligible and does not affect the performance of the algorithm.

To increase the efficiency of sMCL-WMR, we include a pre-calculated table storing *μ*_*m*_, *μ*_*d*_,  and  for common values of ℓ, *d*, and *n *(for examples see Table 1). We varied *n *to be between 10 and 50, ℓ to be between 15 and 30, and *d *to be between ⌊ℓ/5⌋ and ⌊ℓ/2⌋ Values with weaker motifs or with small data sets (*i.e*. when *n *≤ 10) are not considered since it was shown that MCL-WMR performs efficiently for these instances [10].

**Table 1:** Weight distribution properties.

(ℓ, *d*)	*μ*_*m*_	*μ*_*d*_			*α*_*m*_	*α*_*d*_	*n*	*μ*_*m*_	*μ*_*d*_			*α*_*m*_	*α*_*d*_
(15, 4)	794	1439	84	84	989	1243	15	432	980	52	60	552	840
(16, 5)	850	1651	86	102	1050	1413	20	794	1439	84	84	989	1243
(18, 6)	899	2204	89	140	1106	1878	25	1529	2250	129	110	1829	1994
(25, 8)	954	2670	111	175	1212	2262	30	1845	3263	196	169	2300	2869
(28, 9)	1024	3230	152	199	1378	2767	35	2240	4523	246	213	2812	4027
(30, 11)	1069	3882	169	245	1462	3312	40	3709	6110	389	275	4613	5460

Data illustrating the change to the mean and standard deviation of the weight of a random motif set and the weight of a random decoy set as the values of ℓ, *d*, and *n *increase. On the left the number of strings is fixed at 20 and on the right the values (ℓ, d) are fixed at (15, 4).

## Results and discussion

### Performance of sMCL-WMR on synthetic data

We follow the experimental methods of Pevzner and Sze [1], and Buhler and Tompa [4] by considering the performance of sMCL-WMR in comparison to other contemporary and well-known motif recognition programs on synthetic data. We fix *n *to be equal to 20, *m *to be 600, and consider varied values of ℓ and *d*. To produce random motif recognition instances, we generate a random motif consensus of length ℓ, then generate *n *occurrences of the motif, each generated from the consensus by randomly choosing *d *positions and for each of the *d *positions choosing a random replacement base from the four possible bases (A, C, G, T). We construct *m *background strings of length *n *and insert the generated motifs into a random position in the string. For each of the (ℓ, *d*) combinations, 100 randomly generated sets of input strings (*n *= 20, *m *= 1000) were generated. The implementation of sMCL-WMR is in C++.

We note that all experimental tests were performed on a Linux machine with a 64-bit 2600 MHz processor and 1 Gbyte of RAM running Ubuntu. We compared the performance of sMCL-WMR with that of the following motif recognition programs: PROJECTION [4], MCL-WMR [10], PMSprune [8], and Voting [9]. All programs were run on the same Linux machine with the same data sets. These motif recognition programs were chosen for their availability, performance, and widespread use; they are appropriate for comparison with sMCL-WMR because of the previously described capability in solving weak motif instances and because of their availability to be run on the described machine. The results of Voting, PMSprune, and PROJECTION are similar to the ones reported by Davila *et al*. [8], and to Chin and Leung [12], both of whose testing was completed on a machine with a slightly slower processor and the same core memory size.

We define the *success rate *of a given program using the performance coefficient used by Pevzner and Sze [1], Buhler and Tompa [4], and others [9,12]. Let *K *denote the set of *t*ℓ base positions in the *t *occurrences of the planted motif, and let *P *denote the corresponding set of base positions in the *t *occurrences predicted by an algorithm. The algorithm's success rate is defined as |*K *∩ *P*|/|*K *∪ *P*|. Table 2 illustrates the comparison between the running time of sMCL-WMR and that of the other programs. Our aim was to test the selected programs on their capability to solve challenging motif instances (*i.e*. when *d *is significantly large with respect to ℓ). In Table 2 "-" implies that the program was not capable of solving the motif instance on the described machine in a reasonable amount of time, which we define to be at most 20 hours, or with reasonable accuracy, which we define to be at least 75%. Two significant trends are witnessed in the data: sMCL-WMR is capable of solving very hard instances of motif recognition (*i.e*. when ℓ = 30 and *d *= 9) and gives a dramatic improvement over the existing programs for instances where ℓ ≥ 14 (for instances where ℓ ≤ 12 sMCL-WMR had comparable or better performance to the other programs). We note that all programs except PROJECTION achieved a 100% success rate on all motif instances; in Table 2 we put the success rate of PROJECTION in brackets.

**Table 2:** Performance on synthetic data with varying (ℓ, *d*).

(ℓ, *d*)	sMCL-WMR	MCL-WMR	PROJECTION	Voting	PMSprune
(10, 2)	15	1020	56 (98%)	< 1	12
(12, 3)	24	2780	321 (85%)	28.4	23
(14, 4)	98	3120	658 (75%)	412	102
(16, 5)	253	4101	1312 (80%)	1620	520
(18, 6)	632	10202	2200 (85%)	4210	33560
(20, 7)	1203	-	2700 (75%)	20021	-
(25, 9)	1502	-	-	-	-
(28, 12)	1691	-	-	-	-
(30, 14)	2002	-	-	-	-

Comparison of the performance of sMCL-WMR and other motif recognition programs on synthetic data; other programs tested include PROJECTION [4], MCL-WMR [10], Voting [9], and PMSprune [8]. All programs except PROJECTION had a success rate of 100% and for this reason, the success rate was for PROJECTION is included in brackets in the table. The time is given in CPU seconds. In all experiments, *n *= 600, *m *= 20, and ℓ and *d *are varied. "-" denotes that the program was not capable of solving the specific problem.

There exist real-genomic data sets which contain a large number of sequences. For example, a data set, labeled as hm20, in the TRANSFAC database [20] has 34 input strings. Unfortunately, it is uncommon to test motif recognition programs with synthetic data sets with greater than 20 input strings. For example, the following motif recognition algorithms were tested with data sets with at most 20 strings: PROJECTION [4], Winnower [1], MITRA [6], PSM1 [7], PMSprune [8], the Voting algorithm [9], MCL-WMR [10], and VAS [12]. We aim to investigate the capability of sMCL-WMR - as well as other motif recognition programs - in solving motif recognition instances with a large number of strings. The other programs tested include MCL-WMR, Voting, and PMSprune. Table 3 shows that sMCL-WMR was capable of solving instance with up to 40 strings. Again, as in Table 2 "-" implies that the program was not capable of solving the motif instance on the described machine in a reasonable amount of time, which we define to be at most 20 hours, or with reasonable accuracy, which we define to be at least 75%. The capability of sMCL-WMR in solving motif recognition instances with a large number of strings can easily be explained by the fact that the runtime of the method used to solve Consensus String scales slowly in *n *and therefore, has efficient running time even when *n *is large (*i.e*. *n *= 40).

**Table 3:** Performance on synthetic data with varying *n*.

*n*	sMCL-WMR	MCL-WMR	PROJECTION	Voting	PMSprune
18	223	5320	698 (85%)	3930	37020
20	243	12032	729 (77%)	5201	45030
24	1354	36112	874 (75%)	10211	-
28	1960	-	-	-	-
30	2504	-	-	-	-
40	3203	-	-	-	-

The performance of sMCL-WMR as the number of strings increases in comparison to other motif recognition programs. Other programs tested include MCL-WMR [10], PROJECTION [4], Voting [9], and PMSprune [8]. The time is given in CPU seconds. In all experiments, ℓ = 18, *d *= 6, *m *= 600 and *n *ranges from 18 to 40.

### Using sMCL-WMR to find regulatory elements

An important biological challenge is to identify DNA binding sites of transcription factors. In this section, we demonstrate the use of sMCL-WMR in discovering these DNA string "motifs" in data sets with a large number of DNA strings. Tompa *et al*. extensively assess 13 motif recognition tools [2] using test sets that make use of transcription factor binding sites. The binding sites were obtained from the TRANSFAC database [20] which contains only eukaryotic transcription factors. The TRANSFAC database is extremely comprehensive, containing data from a large variety of species, including yeast, *mus*, *oryctolagus cuniculus*, and *homo sapiens *[20]. For more details concerning the data set, including the selection process for transcription factors and binding sites from TRANSFAC, see Tompa *et al*. [2].

We ran sMCL-WMR on a randomly selected set of set of transcription factors from those of Tompa *et al*. [2]. Each transcription factor gives rise to one set of strings. The number of strings varied from 34 (hm20) to 8 (hm26) and the string length (parameter *m*) varied from 700 bp to 2000 bp. Experimental results are shown in Table 4. sMCL-WMR was capable of discovering motifs for these data sets, as well as many motifs not yet found by the motif recognition programs assessed by Tompa *et al*. [2]. The known binding sites shown in Table 4 are as given by the TRANSFAC database Tompa *et al*. [2].

**Table 4:** Motif recognition on biological data.

Data set	Published motif	Motif pattern discovered	Motif recognition program	ℓ	*d*	Time (CPU sec.)
hm01	gggaggctgaggcatgag	cggaggcctaagcctcag	GLAM [22]	18	8	42.1
hm03	Cagccaggctgcagtgctg	Catccatacagaa	GLAM [22]	13	6	12.3
hm04	gcgatgtgtaatagtcgc	gacatgtgtaaaaga	MEME [11]	15	9	54.2
hm08	ggagaaattctaa	aTGACgTC	Weeder [14]	13	6	4.34
hm20	ctgTAatc	gagTAaac	MITRA [6]	8	3	6.7
hm26	GCCGGC	GCCGGC	MITRA [6]	6	0	3.44

Data collected from TRANSFAC and the published motifs are found by the assessment of Tompa *et. al*. [2] of differing motif tools. For each set of data, we determined motifs of the same length using sMCL-WMR. The number of strings ranges from 8 to 34.

## Conclusion

In this paper we investigate the relationship between the weight of a decoy set and the weight of a motif set by means of random sampling. We discuss a rejection sampling strategy, and propose a means to make this uniform sampling method more efficient. Using our proposed sampling algorithm, we study the probability distributions of the respective weights of a random motif set and a random decoy set. We conclude that the weight of a pairwise bounded set can accurately predict whether the set is a valid motif set; we then use this heuristic to develop a program that efficiently detects motifs in large data sets. Our focus was to develop an efficient program that solves a combinatorial version of the motif recognition problem. A position weight matrix (PWM) is another commonly used representation of motifs in biological strings [21]. The application of techniques described in this paper - graph clustering and satistical thresholds - to the PWM model of motif recognition warrants further investigation.

## Competing interests

The authors declare that they have no competing interests.

## Authors' contributions

Concept, implementation, and experiments: CB. Analysis and manuscript preparation: CB and JK.
